# Supplementary material for: Molecular and Morphological Evidence Reveals Four New Neocosmospora Species from Dragon Trees in Yunnan Province, China
Source: J Fungi (Basel). 2025 Jul 31;11(8):571. doi: 10.3390/jof11080571 (PMC12387650; doi:10.3390/jof11080571)
Supplement: Supplementary file 1 [file jof-11-00571-s001.zip › jof-3580162-supplementary.pdf]

Supplementary Table S1. Specimen information and GenBank accession numbers for sequences used in this study.

Taxon	Host	Voucher	GenBank accession number				
			ITS	nrLSU	tef-1α	rpb1	rpb2
Geejayessia atrofusca	***Staphylea trifolia***	NRRL 22316	AF178423	AF178392	AF178361	JX171496	EU329502
G. cicatricum	Dead twig	CBS 125552	HQ728145	MH875038	HM626644	–	HQ728153
Neocosmospora acutispora	*Coffea arabica*	CBS 145461^{T} = NRRL 22574 = BBA 62213	LR583700	LR583908	LR583593	MW834210	LR583814
N. addoensis	*Citrus sinensis*	CBS 146510^{T} = CPC 37128	MW173042	MW173033	MW248741	MW218098	MW446575
N. akasia	*Euwallacea perbrevis*	CMW 54735^{T} = PPRI 27978 = CBS 146880	MN954357	MN954357	MT009951	–	MT009931,MT010011
N. ambrosia	*Euwallacea fornicatus*	CBS 571.94^{T} = NRRL 22346 = BBA 65390 = MAFF 246287	EU329669	EU329669	FJ240350	MW834211	EU329503
N. ampla	*Coffea sp.*	BBA 4170^{T} = CBS 202.32	LR583701	LR583909	LR583594	MW834212	LR583815
N. anhuiensis	*Rotten twigs*	CGMCC 3.24869^{T}	OQ842733	–	OQ866530	–	OQ866525
N. aquatica	Wood	KUNCC 22–12459^{T} =CGMCC 3.24275	OP876713	OP875069	OQ064518	–	–
N. aurantia	Rotten bark	CGMCC 3.24866^{T}	OQ842731	–	OQ866528	–	OQ866523
N. awan	*Euwallacea similis*	CMW 54719^{T} = PPRI 27973 = CBS 146882	MN954345	MN954345	MT009973	–	MT009919,MT009999
N. bataticola	*Ipomoea batatas*	NRRL 22402^{T}=BBA 64954 = CBS 144398 = FRC S-0567	AF178408	AF178377	AF178344	MW218100	FJ240381
N. bomiensis	Twigs	HMAS 248885^{T}	KY829447	–	KY829449	–	–
N. borneensis	Bark or recently dead tree	CBS 145462^{ET} = NRRL 22579 = BBA 65095	AF178415	AF178384	AF178352	MW834213	EU329515
N. bostrycoides	Soil	CBS 144.25^{NT}	LR583704	LR583912	LR583597	MW218101	LR583818
N. brasiliense	*Glycines max*	NRRL 31757^{T}	AY320184	AY320130	AY320148	–	EU329565
N. brevicona	*Gladiolus sp.*	BBA 2123^{ET} = CBS 204.31 = NRRL 22659	LR583707	MH866639	LR583600	MW218103	LR583821
N. brevis	Polluted soilwater	CBS 144387^{T} = MUCL 16108	LR583708	–	LR583601	MW834214	LR583822
N. bugnicourtii	*Euwallacea fornicatus on Camellia sinensis*	IMI 296597^{T} = NRRL 20438 = MAFF 246291	AF178397	AF178366	AF178332	JX171470	JX171584
N. caricae	*Ficus carica*	CBS 148865^{T}	OK422518	–	OK539518	–	OK415859
N. catenata	*Stegostoma fasciatum*	CBS 143229^{T} = NRRL 54993	KC808256	KC808256	KC808214	KC808292	KC808355
N. citricola	*Citrus sinensis*	CBS 146513^{T} = CPC 37131	MW173048	MW173036	MW248747	MW218108	MW446581

Taxon	Host	Voucher	GenBank accession number				
			ITS	nrLSU	tef-1α	rpb1	rpb2
N. crassistipitatum	*Glycine max*	NRRL 36877^{T}	–	FJ240376	FJ240351	–	FJ240405
N. croci	*Citrus sinensis*	CBS 142423^{T} = CPC 27186	LT746264	–	LT746216	–	LT746329
N. cryptoseptata	Bark	BBA 65024^{T} = CBS 145463 = NRRL 22412	AF178414	AF178383	AF178351	MW834215	EU329510
N. cucurbitae	*Cucurbita viciifolia*	CBS 616.66^{T} = NRRL 22399 = BBA 64411	LR583711	LR583919	DQ247592	MW834217	LR583825
N. cryptoseptata	Bark	BBA 65024^{T} = CBS 145463 = NRRL 22412					
N. cuneirostrum	*Vigna angularis*	NRRL 31157	EF408519	AY320142	AY320160	KJ511271	FJ240389
N. cyanescens	Human foot	CBS 518.82^{T}	AB190389	LR583920	LR583605	MW218110	LR583826
N. dimiuta	*Coelocaryon preussii*	CBS 144390^{T} = MUCL 18798	LR583713	LR583922	LR583607	MW834218	LR583828
N. dimorpha	Rotten twigs	CGMCC 3.24867^{T}	OQ842732	–	OQ866529	–	OQ866524
N. drepaniformis	Woody Plantae	NRRL 62941^{T}	KM406633	KM406633	KM406626	KM406640	KM406647
N. duplosperma	*Euwallacea perbrevis*	NRRL 62583^{T}	KC691581	KC691581	KC691553	KC691611	KC691642
N. elegans	*Xanthoxylum piperitum*	CBS 144396^{ET} = NRRL 22277 = MAFF 238541 = ATCC 42366	AF178401	AF178370	AF178336	MW218113	FJ240380
N. epipeda	*Bouvardia sp.* imported from Uganda	CBS 146523^{T} = CPC 38310	MW827624	MW827665	MW834285	MW834219	MW834022
N. euwallaceae	*Euwallacea sp.*	CBS 135854^{T} = NRRL 54722	JQ038014	JQ038014	JQ038007	JQ038021	JQ038028
N. falciformis	Human mycetoma	CBS 475.67^{T} = IMI 268681	MG189935	MG189915	LT906669	MW218114	LT960558
N. ferruginea	Human subcutaneous nodule	CBS 109028^{T} = NRRL 32437	DQ094446	DQ236488	DQ246979	HM347157	EU329581
N. floridana	*Euwallacea interjectus*	NRRL 62628^{T} = MAFF 246849	KC691563	KC691563	KC691535	KC691593	KC691624, KC691653
N. galbana	Bark	CGMCC 3.24868^{T}	OQ842730	–	OQ866532	–	OQ866527
N. gamsii	Human bronchoalveolar lavage fluid	CBS 143207^{T} = NRRL 32323 = UTHSC 99-205	DQ094420	DQ236462	DQ247103	MW834223	EU329622
N. gamtoosensis	*Citrus sinensis*	CBS 146502^{T} = VG16 = CPC 37120	MW173063	MW173038	MW248762	MW218116	MW446611
N. gannanensis	*Euwallacea interjectus*	NJFU-JX12^{T}	LC702041	LC702041	LC701609	LC701945	LC701981
N. geoasparagicola	Soil	CBS 148937^{T}	ON763207	–	ON745622	ON759290	ON759301
N. haematococca	Dying tree	CBS 119600^{ET}=FRC S-1832	KM231797	KM231664	KM231926	KM232216	LT960561
N. hengyangensis	Twigs	HMAS 254518	KY829446	–	KY829448	–	–
N. hypertrophia	*Dracaena cochinchiesis*	YIM F00427^{T}	OR687704	OR687699	PP505835	PP400446	PP457517

Taxon	Host	Voucher	GenBank accession number				
			ITS	nrLSU	tef-1*α*	rpb1	rpb2
N. hypertrophia	*Dracaena cochinchiesis*	YIM_F00403	OR687706	OR687701	PP505837	PP400448	PP457519
N. hypertrophia	*Dracaena cochinchiesis*	YIM_F00422	OR687707	OR687702	PP505836	PP400447	PP457518
N. hypertrophia	*Dracaena cochinchiesis*	YIM_F00490	OR687705	OR687700	PP505833	PP316622	OR962074
N. hypertrophia	*Dracaena cochinchiesis*	YIM_F00497	OR687708	OR687703	PP505834	PP400445	PP437595
N. hypothenemi	*Hypothenemus hampei*	CBS 145464^{T} = NRRL 52782 = ARSEF 5878	LR583715	LR583923	JF740850	MW218117	JF741176
N. illudens	*Beilschmiedia tawa*	CBS 147303 = NRRL 22090 = BBA 67606 = GJS 82- 98	AF178393	AF178362	AF178326	JX171488	JX171601
N. ipomoeae	*Gerbera sp.*	CBS 353.87 = NRRL 22657	LR583717	LR583925	DQ247639	MW218119	LR583831
N. keleraja	Branch of unidentified tree	CBS 125722^{PT} = FRC S-1836 = GJS 02-114	JF433039	JF433039	DQ247515	MW834226	LR583835
N. keratoplastica	Human eye	CBS 490.63^{T}	LR583721	LR583929	LT906670	MW218121	LT960562
N. kunmingense	*Dracaena cochinchiesis*	YIM F00502^{T}	OR681902	OR681906	OR670497	OR670501	OR670503
N. kunmingense	*Dracaena cochinchiesis*	YIM F00315	OR681905	OR681909	OR670495	OR670499	
N. kunmingense	*Dracaena cochinchiesis*	YIM F00361	OR681903	OR681907	OR670498	OR670502	OR670504
N. kunmingense	*Dracaena cochinchiesis*	YIM F00373	OR681904	OR681908	OR670496	OR670500	OR670505
N. kuroshio	*Platanus racemosa*	CBS 142642^{T} = UCR 3641	LR583723	LR583931	KX262216	KX262236	LR583837
N. kurunegalensis	Recently felled tree	CBS 119599^{T}= GJS 02-94=FRC S-1833	JF433036	JF433036	DQ247511	MW834228	LR583838
N. lerouxii	*Citrus sinensis*	CBS 146514^{T} = CPC 37132	MW173069	MW173039	MW248768	MW218123	MW446617
N. lichenicola	*Homo sapiens*	CBS 623.92^{ET}	LR583730	LR583938	LR583620	–	LR583845
N. liriodendri	*Liriodendron tulipifera*	CBS 117481^{T} = NRRL 22389 = BBA 67587 = GJS 91- 148	AF178404	AF178373	AF178340	MW218124	EU329506
N. lithocarpi	*Lithocarpus glabra*	LC1113	MW016711	–	MW620172	MW024739	MW474697
N. liupanshuiense	*Rosa roxburghii*	GUCC 190201.1^{T} = CGMCC3.25481	MZ724850	OR039401	OR043924	OR043815	–
N. longissima	Tree bark	CBS 126407^{T} = G.J.S. 85-72	LR583731	LR583939	LR583621	MW834230	LR583846
N. macrospora	*Citrus sinensis*	CBS 142424^{T} = CPC 28191	LT746266	LT746281	LT746218	MW218125	LT746331
N. magnoliae	*Magnolia champaca*	MFLUCC 17-2615^{T}	MT215508	MT215557	MT212207	–	MT212200
N. mahasenii	Live tree	CBS 119594^{T} = FRC S-1845	JF433045	JF433045	DQ247513	MW834231	LT960563
N. maoershanica	Twigs	CGMCC 3.24870^{T}	OQ842734	–	OQ866531	–	OQ866526
N. martii	*Solanum tuberosum*	CBS 115659^{ET} = FRC S-0679 = MRC 2198	JX435206	JX435206	JX435156	MW834232	JX435256

Taxon	Host	Voucher	GenBank accession number				
			ITS	nrLSU	tef-1α	rpb1	rpb2
N. mekan	*Euwallacea similis*	CMW 54714^{T} = PPRI 27971 = CBS 146885	MN954342	MN954342	MT009964	–	MT009916,MT009996
N. merkxiana	*Chrysanthemum sp.* imported from Uganda	CBS 146525^{T}	MW827627	MW827668	MW834288	MW834233	MW834025
N. metavorans	Pleural effusion	CBS 135789^{T}	LR583738	LR583946	LR583627	MW218127	LR583849
N. mori	*Solanum tuberosum*	CBS 145467^{T} = NRRL 22230 = MAFF 238539	DQ094305	DQ236347	AF178358	MW834235	EU329499
N. neerlandica	*Pisum sativum*	CBS 232.34^{T}	MW827629	MW827670	MW847906	MW834237	MW847903
N. nelsonii	*Pisum sativum*	CBS 309.75^{T}	MW827630	MW827671	MW847907	MW834238	MW847904
N. nirenbergiana	Bark	CBS 145469^{T} = NRRL 22387 = BBA 65023 = GJS 87-127	AF178403	AF178372	AF178339	–	EU329505
N. noneumartii	*Solanum tuberosum*	CBS 115658^{T} = FRC S-0661	LR583745	LR583949	LR583630	MW218129	MW446618
N. oblonga	Human eye	CBS 130325^{T}= NRRL 28008 = CDC B-4701	LR583746	LR583950	LR583631	MW834239	LR583853
N. pisi	Progeny of parentals from *Pisum sativum* and soil	NRRL 45880^{ET} =CBS 123669 =ATCC MYA-4622	FJ240312	LR583957	FJ240352	MW834242	LR583862
N. plagianthi	*Hoheria glabrata*	NRRL 22632= GJS 83-146	AF178417	AF178386	AF178354	JX171501	JX171614
N. populicola	*Euwallacea interjectus*	NJFU-JS02^{T}	LC702061	LC702061	LC701601	LC701929	LC701965, LC702001
N. protoensiformis	Bark of dicot tree	CBS 145471^{T} = NRRL 22178 = GJS 90-168	AF178399	AF178368	AF178334	MW834244	EU329498
N. pseudensiformis	Bark of tree	CBS 125729^{T} = FRC S-1834 = G.J.S 9318 = NRRL 46517	KC691584	KC691584	KC691555	KC691615	KC691674
N. pseudopisi	*Pisum sativum*	CBS 266.50	MW827631	MW827672	MW834290	MW834246	MW834027
N. pseudoradicicola	*Theobroma cacao*	CBS 145472^{T} = NRRL 25137	JF740899	JF740899	JF740757	MW218133	JF741084
N. pseudotonkinensis	Human cornea	CBS 143038	MG189942	MG189927	LR583640	–	LR583867
N. quercicola	*Quercus cerris*	CBS 141.90^{T} = NRRL 22652	LR583760	LR583964	DQ247634	MW834247	LR583869
N. rectiphora	Bark	CBS 125727^{T}=GJS 02-89 = FRC S-1831	JF433034	JF433034	LR583641	MW834249	LR583871
N. regularis	*Pisum sativum*	CBS 230.34^{T}	LR583763	LR583967	LR583643	–	LR583873
N. rekana	*Acacia crassicarpa*	CMW 52862^{T} = PPRI 27163	MN249094	–	MN249151	–	MN249137, MN249108
N. riograndensis	Human nasal cavity	UFMG-CM F12570^{T} = URM-7361	KT186366	KX534001	KX534002	–	KX534003

Taxon	Host	Voucher	GenBank accession number				
			ITS	nrLSU	tef-1*α*	rpb1	rpb2
N. robusta	Bark	CBS 145473^{T} = NRRL 22395 = BBA 65682	AF178405	LR583968	AF178341	MW834251	EU329507
N. rubicola	Soil	CBS 101018^{T}	KM231800	KM231667	KM231928	KM232219	KM232367
N. samuelsii	Bark	CBS 114067^{T} = G.J.S. 89-70	LR583764	LR583969	LR583644	MW834252	LR583874
N. sedimenticola	Deep-sea sediments	CGMCC 3.19499^{T} = LC12845	MK207059	–	MK190727	–	MK190729
N. silvicola	*Liriodendron tulipifera*	CBS 123846^{T} = G.J.S. 04-147	LR583766	LR583971	LR583646	MW834254	LR583876
N. simplicillium	*Dracaena cochinchiesis*	YIM F00563^{T}	OR687709	PP419963	PP238905	PP400450	PP485046
N. simplicillium	*Dracaena cochinchiesis*	YIM F00656	OR687710	PP419964	PP133248	PP400449	PP485045
N. simplicillium	*Dracaena cochinchiesis*	YIM F00566	OR687711	PP419965	PP238904	PP273273	PP485044
N. solani	*Solanum tuberosum*	CBS 140079^{ET} = NRRL 66304 = GJS09-1466 = FRC S-2364	KT313633	KT313633	KT313611	MW218134	KT313623
N. spathulata	Human synovial fluid	CBS 145474^{T} = NRRL 28541	EU329674	EU329674	DQ246882	MW218137	EU329542
N. stercicola	Compost yard debris	CBS 142481^{T}=DSM 106211	LR583779	LR583984	LR583658	MW834255	LR583887
N. striatispora	Dicotyledonous plant	MFLU:19-0975^{T}	ON775553	–	ON892545	–	–
N. suttoniana	Human wound	CBS 143214^{T} = NRRL 32858	DQ094617	DQ236659	DQ247163	MW218138	EU329630
N. thailandica	Dead stem	MFLU:18-2712^{T}	ON787624	ON787612	ON892546	–	–
N. tonkinensis	*Musa sapientum*	CBS 115.40^{T}	MG189941	MG189926	LT906672	MW218140	LT960564
N. tuaranensis	*Hevea brasiliensis*	NRRL 22231^{T} = ATCC 16563 = MAFF 246842	KC691570	KC691570	KC691542	KC691600	KC691631
N. tucumaniae	*Glycine max*	NRRL 31096^{T} = MAFF 238418 = MJ-172	GU170656	AY220161	GU170636	–	GU170616
N. tumidisperma	*Euwallacea interjectus*	NJFU-JX26^{T}	LC702053	LC702053	LC701621	LC701957	LC701993, LC702029
N. variasi	*Acacia crassicarpa*	CMW 53734^{T} = PPRI 27958 = CBS 146888	MN954356	MN954356	MT009967	–	MT009913,MT009993
N. vasinfecta	*Gossypium sp.*	ATCC 62199= NRRL 22166	–	–	AF178350	–	EU329497
N. vasinfecta	*Heterodera glycines*	CBS 325.54 = ATCC 16238 = IFO 7591 = IMI 251386 = NRRL 22436	AF178412	AF178381	AF178348	JX171497	JX171610
N. virgulifore	*Glycine max*	NRRL 31041^{T}	AY220239	–	AY220193	JX171530	JX171643
N. witzenhausenense	*Hibiscus sp.*	CBS 142480^{T} = DSM 106212	MG250477	MG250478	KY556525	MG237865	LR583886

Taxon	Host	Voucher	GenBank accession number				
			ITS	nrLSU	tef-1α	rpb1	rpb2
N. wrinkles	*Dracaena cochinchiesis*	YIM F00493[T]	OR681554	OR681558	OR733715	OR733716	OR733717
N. xiangyunense	Waterlogged soil	CGMCC 3.19676	MH78092 3	–	MH99262 9	MH99928 1	

ATCC: American Type Culture Collection, Manassas, VA, USA; BBA: Biologische Bundesanstalt für Land- und Forstwirtschaft, Institut für Mikrobiologie, Berlin, Germany; CDC: Centers for Disease Control and Prevention, Atlanta, GA, USA; CPC: Collection of P.W. Crous, held at WI; DSM: DSMZ-Deutsche Sammlung von Mikroorganismen und Zellkulturen GmbH, Braunschweig, Germany; FRC: Fusarium Research Center, Pennsylvannia State University, PA, USA; HMAS: Herbarium Mycologicum Academiae Sinicae, Chinese Academy of Sciences, Beijing, China; IMI: CABI Bioscience, Eggham, UK; MAFF: Ministry of Agriculture, Forestry and Fisheries, Tsukuba, Ibaraki, Japan; MRC: National Research Institute for Nutritional Diseases, Tygerberg, South Africa; MUCL: Mycothèque de l´Université Catholique de Louvain, Louvain-la-Neuve, Belgium; NRRL: Agricultural Research Service Culture Collection, National Center for Agricultural Utilization Research, USDA, Peoria, IL, USA; UFMG: Coleção de Micro-organismos, DNA e Células da Universidade Federal de Minas Gerais, Belo Horizonte, Brazil; UTHSC: Fungus Testing Laboratory, Department of Pathology, University of Texas Health Science Center, San Antonio, USA. YIM: Yunnan Microbiological Institute, School of Life Science, Yunnan University. ET: Ex-epitype, NT: Ex-neotype, PT: Ex-paratype; T: Ex-type; – : represents the absence of sequence data in GenBank.
